# The cholera risk assessment in Kano State, Nigeria: A historical review, mapping of hotspots and evaluation of contextual factors

**DOI:** 10.1371/journal.pntd.0009046

**Published:** 2021-01-19

**Authors:** Moise Chi Ngwa, Chikwe Ihekweazu, Tochi Okwor, Sebastian Yennan, Nanpring Williams, Kelly Elimian, Nura Yahaya Karaye, Imam Wada Bello, David A. Sack

**Affiliations:** 1 Department of International Health, Johns Hopkins Bloomberg School of Public Health, Baltimore, Maryland, United States of America; 2 Nigeria Centre for Disease Control, Abuja, Nigeria; 3 Department of Microbiology, University of Benin, Nigeria; 4 Department of Public Health and Disease Control, Kano State Ministry of Health, Kano, Nigeria; 5 Department of Public Health and Disease Control, Ministry of Health Kano, Kano, Nigeria; Faculty of Medicine and Health Sciences, Universiti Putra Malaysia, MALAYSIA

## Abstract

Nigeria is endemic for cholera since 1970, and Kano State report outbreaks annually with high case fatality ratios ranging from 4.98%/2010 to 5.10%/2018 over the last decade. However, interventions focused on cholera prevention and control have been hampered by a lack of understanding of hotspot Local Government Areas (LGAs) that trigger and sustain yearly outbreaks. The goal of this study was to identify and categorize cholera hotspots in Kano State to inform a national plan for disease control and elimination in the State. We obtained LGA level confirmed and suspected cholera data from 2010 to 2019 from the Nigeria Centre for Disease Control (NCDC) and Kano State Ministry of Health. Data on inland waterbodies and population numbers were obtained from online sources and NCDC, respectively. Clusters (hotspots) were identified using SaTScan through a retrospective analysis of the data for the ten-year period using a Poisson discrete space-time scan statistic. We also used a method newly proposed by the Global Task Force on Cholera Control (GTFCC) to identify and rank hotspots based on two epidemiological indicators including mean annual incidence per 100 000 population of reported cases and the persistence of cholera for the study period. In the ten-year period, 16,461 cholera cases were reported with a case fatality ratio of 3.32% and a mean annual incidence rate of 13.4 cases per 100 000 population. Between 2010 and 2019, the most severe cholera exacerbations occurred in 2014 and 2018 with annual incidence rates of 58.01 and 21.52 cases per 100 000 inhabitants, respectively. Compared to 2017, reported cases and deaths increased by 214.56% and 406.67% in 2018. The geographic distribution of outbreaks revealed considerable spatial heterogeneity with the widest in 2014. Space-time clustering analysis identified 18 out of 44 LGAs as high risk for cholera (hotspots) involving both urban and rural LGAs. Cholera clustered around water bodies, and the relative risk of having cholera inside the hotspot LGA were 1.02 to 3.30 times higher than elsewhere in the State. A total of 4,894,144 inhabitants were in these hotspots LGAs. Of these, six LGAs with a total population of 1.665 million had a relative risk greater than 2 compared to the state as a whole. The SaTScan (statistical) and GTFCC methods were in agreement in hotspots identification. This study identified cholera hotspots LGAs in Kano State from 2010–2019. Hotspots appeared in both urban and rural settings. Focusing control strategies on these hotspots will facilitate control and eliminate cholera from the State.

## Author summary

In Nigeria, cholera occurs annually since 1970 and Kano State located in the North West zone of the country reports cholera outbreaks almost every year. Although both national and Kano State governments provide water, sanitation, and hygiene interventions to stop the outbreaks, the disease still occurs in many Local Government Areas. With this continuous threat from cholera in Kano State annually, we used Local Government Area level suspected and confirmed cholera data from 2010 to 2019 to identify and classify ‘hotspot’ Local Government Areas. Our goal was to better guide National Cholera Plans for control and elimination of cholera from Kano State. In this ten-year period, there were 16,461 cholera cases with annual incidence rate of 13.4 cases per 100 000 people. A population of 4,894,144 people live in the 18 cholera hotspots Local Government Areas in which cholera risks were 1.02 to 3.30 times higher than elsewhere in Kano State. As recommended by Global Task Force for Cholera Control, interventions should focus on these identified hotspots Local Government Areas to facilitate control and elimination of cholera from Kano State.

## Introduction

Cholera threatens public health worldwide making over a million people sick with an estimated 90,000 deaths yearly [1]. However, sub-Saharan Africa (SSA) [2] has the highest burden second to Asia as the leading region for cholera cases and deaths between 2014 and 2018 [3–7] with Nigeria reporting the highest cases of cholera in SSA in 2018 [7]. The disease appeared in Nigeria in 1970 and gravitated towards an endemic pattern with huge outbreaks reported in 1991 (59,478 cases, case fatality ratio (CFR) 13%)), 2010 (44,556, CFR 4%), and 2014 (35,996 cases, CFR 2%) [8]. In 2018, epidemiological surveillance reported 42,466 cases (CFR 2%) [9], representing a 240% increase in cases compared to 2017. Broadly, cholera occurs in areas with sub-optimal water, sanitation and hygiene (WASH) systems, but there is minimal understanding of the epidemiology and ecology of transmission patterns needed to inform intervention strategies [10].

**Fig 1:**
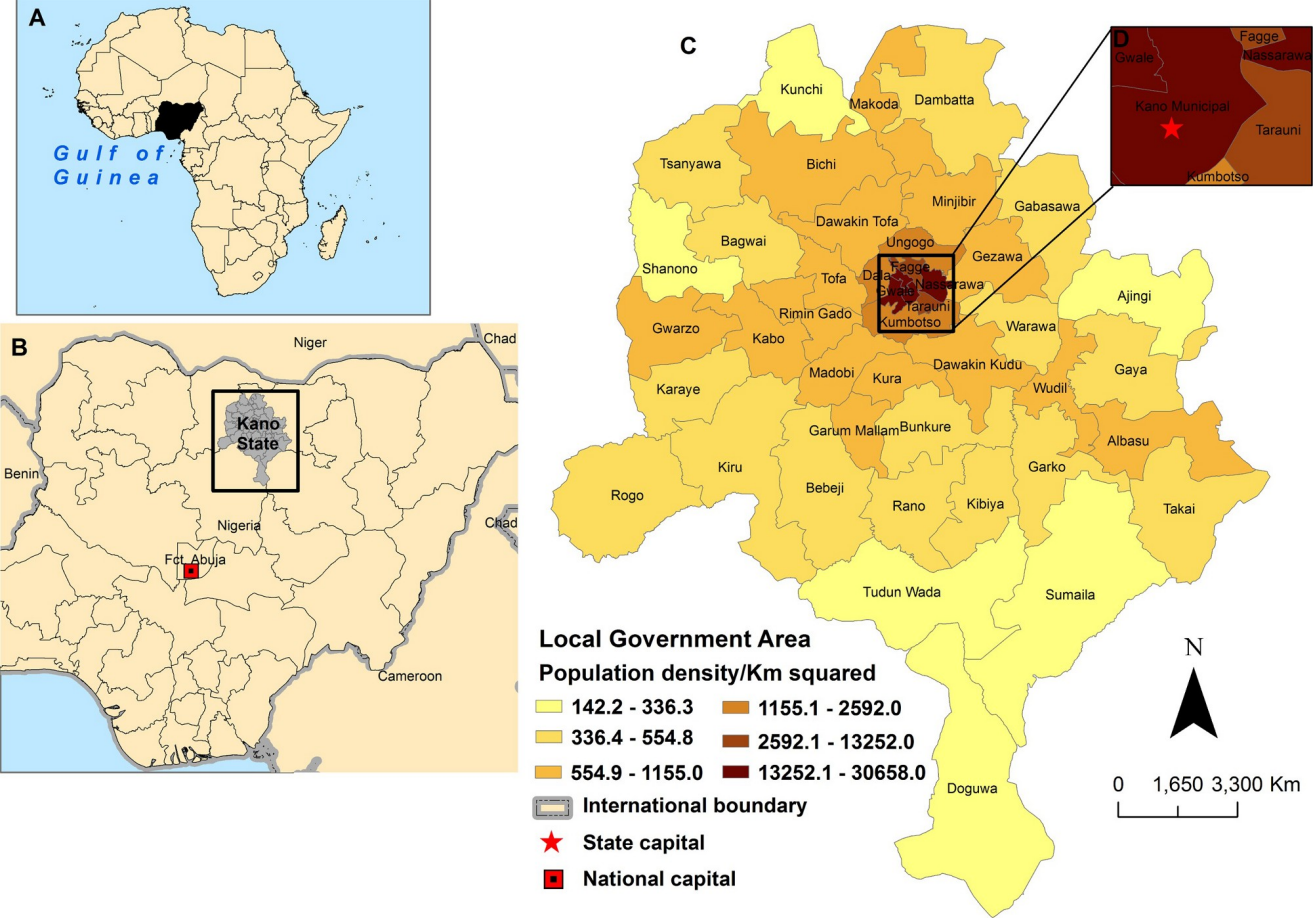
Map of our study setting describes the population density distribution of Kano State. Insert **(A)** shows Nigeria within Africa while **(B)** depicts Nigeria capital Abuja and Kano State within Nigeria. Insert **(C)** depicts population density of the 44 Local Government Areas of Kano State and insert **(D)** shows a better view of Kano Municipal, the capital of Kano State.

Although cholera is endemic in Nigeria (Fig 1A and 1B), attack rates vary between the different states, with Kano State (Fig 1C) reporting cases nearly every year [11]. Statistical modeling of hospital case data from Kano State and other Northern states from 1991 to 2011 demonstrated a marked annual cycle, with peak transmission between April and August [11–14]. Increases in temperature, rainfall, poverty, and population density were found to be associated with both cholera cases and deaths [11]. In 1999, Kano Municipal Local Government Area (LGA), Kano State, reported a cholera outbreak that was traced to the interruption of domestic water supply leading to 815 cases with 28 deaths [15]. The outbreak spread to Tofa LGA (182 cases, 19 deaths) and further to Adamawa (76 cases, 18 deaths) and Edo (49 cases, 24 deaths) states. In early November 2001, 18 out of the 44 LGAs in Kano State reported 2050 cases and 80 deaths while in November 2002, the State reported 176 cases and 12 deaths [15]. Adewale *et al*. [14] analyzed *V*. *cholerae* O1 clinical and environmental (borehole, well, stream, and tap water) isolates from the 2007–2013 cholera outbreaks involving Kano State and eight others using PCR and PFGE (pulsed field gel electrophoresis) techniques in an attempt to understand the characteristics of the circulating strains. Out of 122 isolates, 115 were confirmed as *V*. *cholerae* O1. Genetically, strains from Abia, Bauchi and Kano States were very different from the rest of the states of Nigeria [14]. Spatial epidemiologic studies of cholera in SSA demonstrate that outbreaks occur in hotspots linked with environmental factors namely lakes, rivers and roads [16–21], floods and droughts [22] as well as climatic variables such as rainfall and temperature [17,23]. Although Kano State is considered one of the hotspot states in Nigeria with a high cholera burden [24,25], we found no studies delineating cholera hotspot LGAs linked with environmental or contextual factors of disease transmission to improve precision in identifying specific areas in need of interventions and control measures.

Slow progress in the control of cholera prompted the World Health Organization (WHO) in 2011 to call for comprehensive measures to cholera control including oral cholera vaccines (OCV) [26] along with WASH. In 2017, the Global Task Force on Cholera Control (GTFCC) launched an initiative to end cholera in 20 countries by 2030 [10] and this initiative calls for identifying cholera hotspots, the small areas heavily affected by cholera, where interventions need to focus. In 2018, Nigeria launched the National Strategic Plan of Action on Cholera Control (NSPACC) to pre-emptively vaccinate populations in identified hotspots nationwide between 2018 and 2023 [27]. However, previous attempts to identify cholera hotspots in Nigeria may not be sufficiently precise to target the populations at highest risk [28,29]. Thus, the NSPACC may benefit from the identification of the specific LGAs that most need vaccine and WASH interventions.

With the goal of better understanding the LGAs at highest risk, we carried out a cholera mapping study for Kano State incorporating statistical methods as well as methods recommended by the GTFCC using clinical surveillance data for the years from 2010 to 2019.

## Methods

### Ethics statement

We used anonymized historical dataset aggregated at the LGA level by the Ministry of Health, Kano State. The Health Research Ethics Committee of the Ministry of Health, Kano State provided ethical approval of the study (*Ref*: MOH/Off/797/T.1/2012) as part of ongoing effort to inform a national plan for disease control and elimination in the State. Based on these, the Johns Hopkins University Internal Review Board (IRB) determined that the study does not qualify as human subjects’ research, and so was exempted from IRB review. Based on anonymity of data, verbal inform consent was not obtained.

### Study setting

Geographically, Nigeria with capital Abuja (Fig 1B) has been grouped into six geopolitical zones; North East, North West, North Central, South East, South West, and the South South [30]. Kano State (Fig 1C, our study site) with capital Kano Municipal (Fig 1D) is located in the North West and is the most populous state in Nigeria, with an estimated population of 13,076,900 in 2016 [31]. It is the second largest industrial centre after Lagos with Hausa as the dominant ethnic group. Kano State is divided into 44 LGAs and of these, six are classified as urban and the rest rural. The average population of an LGA in Kano State is about 300,000 [32]. The population density is highest in the Central urban LGAs and progressively decreases outwards towards the rural LGAs with a range of 142.2 km^2^ in Doguwa to 30,658 km^2^ in Dala (Fig 1C). The 44 LGAs are further divided into 484 wards (administrative unit level four) with about 10 to 15 wards per LGA. This state was chosen because of the interest of public health officials (NCDC and Kano Ministry of Health) in cholera control and because of recurrent cholera outbreaks [24,25,33]. In addition, OCV campaigns are planned to be conducted in Kano between 2020 and 2023; and thus, this work will provide data needed to inform decision making on the highest priority LGA hotspots.

### Data collection

We obtained aggregated cholera historical data (both confirmed and suspected cases) at the LGA level on reported morbidity and mortality from the Nigeria Centre for Disease Control (NCDC) and Kano State Ministry of Health for the period from 2010 to 2019. The data were collected guided by cholera case definition [34] within the framework of the health facility based Integrated Disease Surveillance and Response (IDSR) Strategy [34,35]. In brief, cholera confirmed and suspected cases are collected at health facility and reported to LGA Disease Surveillance and Notification Officer where data are compiled and sent to the State Epidemiologist at the State Ministry of Health. The state analyzes the data and sends these to the NCDC, the national public health institute that coordinates surveillance activities, and from there to other government entities and international partners. The 2018 Demographic and Health Survey for Nigeria [36]used population denominators derived from the most recent population and housing census for Nigeria in 2006 [37]. As such, we derived population sizes of each LGA from 2010 to 2019 from the 2006 population census [37]; no population census for Nigeria is available after the 2006 census. A population growth rate estimate of 3.3 (obtained from NCDC) for all LGAs within Kano State was used to estimate the annual population sizes for each LGA for 2010–2019. Polygon Shapefiles for the national, state and LGA boundaries were obtained from Public Health Officials from Nigeria and classification of LGAs into urban and rural areas were read from these Shapefiles. Further, we downloaded inland waterbodies (i.e., contextual factors of transmission) from the open source platform DIVA-GIS (http://www.diva-gis.org/Data). All spatially referenced data were projected into the Universal Transverse Mercator, Zone 32N, coordinate system.

### Descriptive spatial and temporal analysis

**Fig 2:**
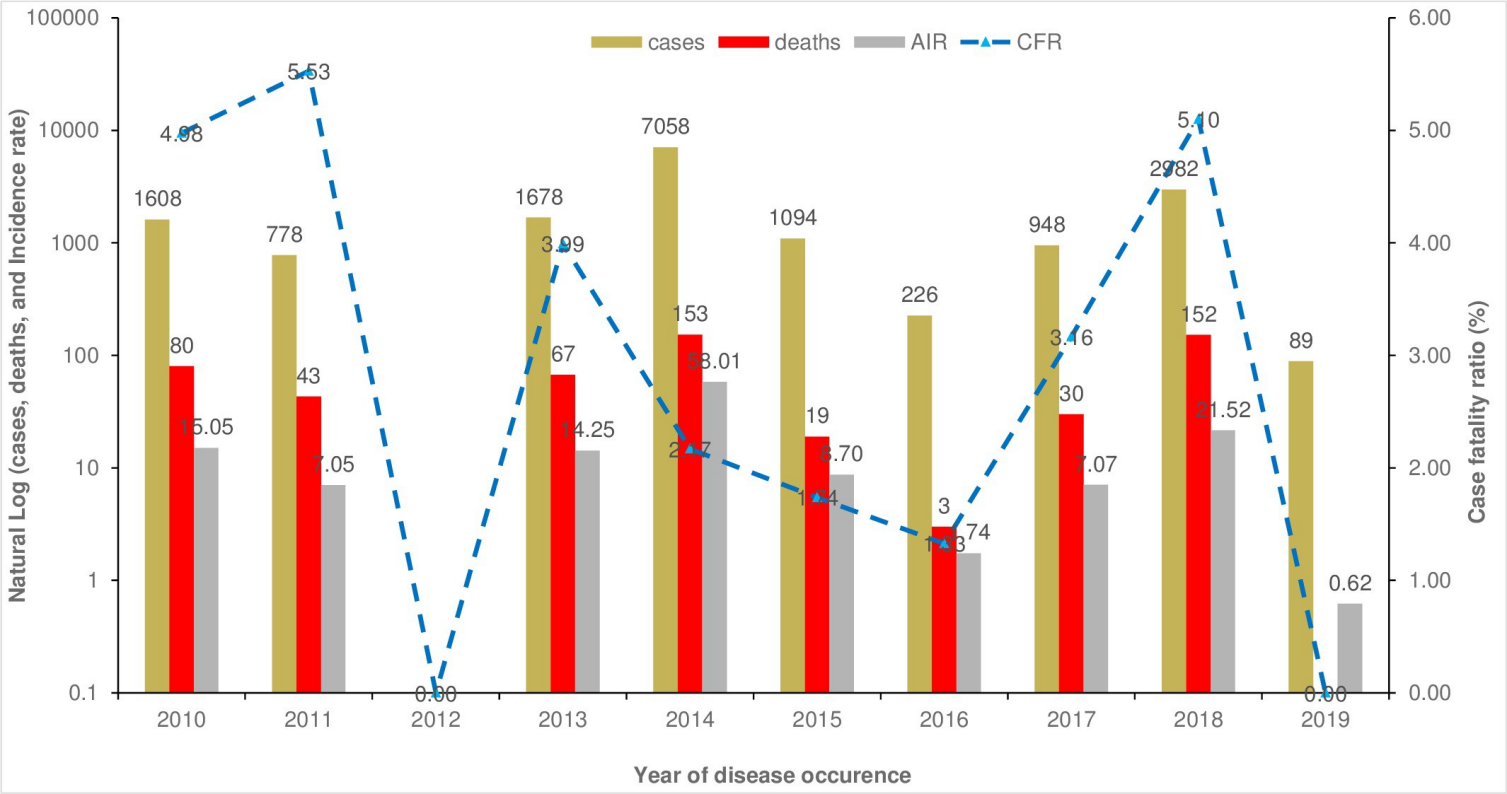
Yearly occurrence of cholera outbreaks in Kano State, Nigeria, from 2010 to 2019. The vertical axis describe cases (brown color), deaths (red color), and annual incidence rate per 100,000-population (grey color) in log scale. The blue dashed line represent case fatality ratio (CFR). There were no reported cholera cases in 2012 while no cholera deaths were reported in 2019.

**Fig 3:**
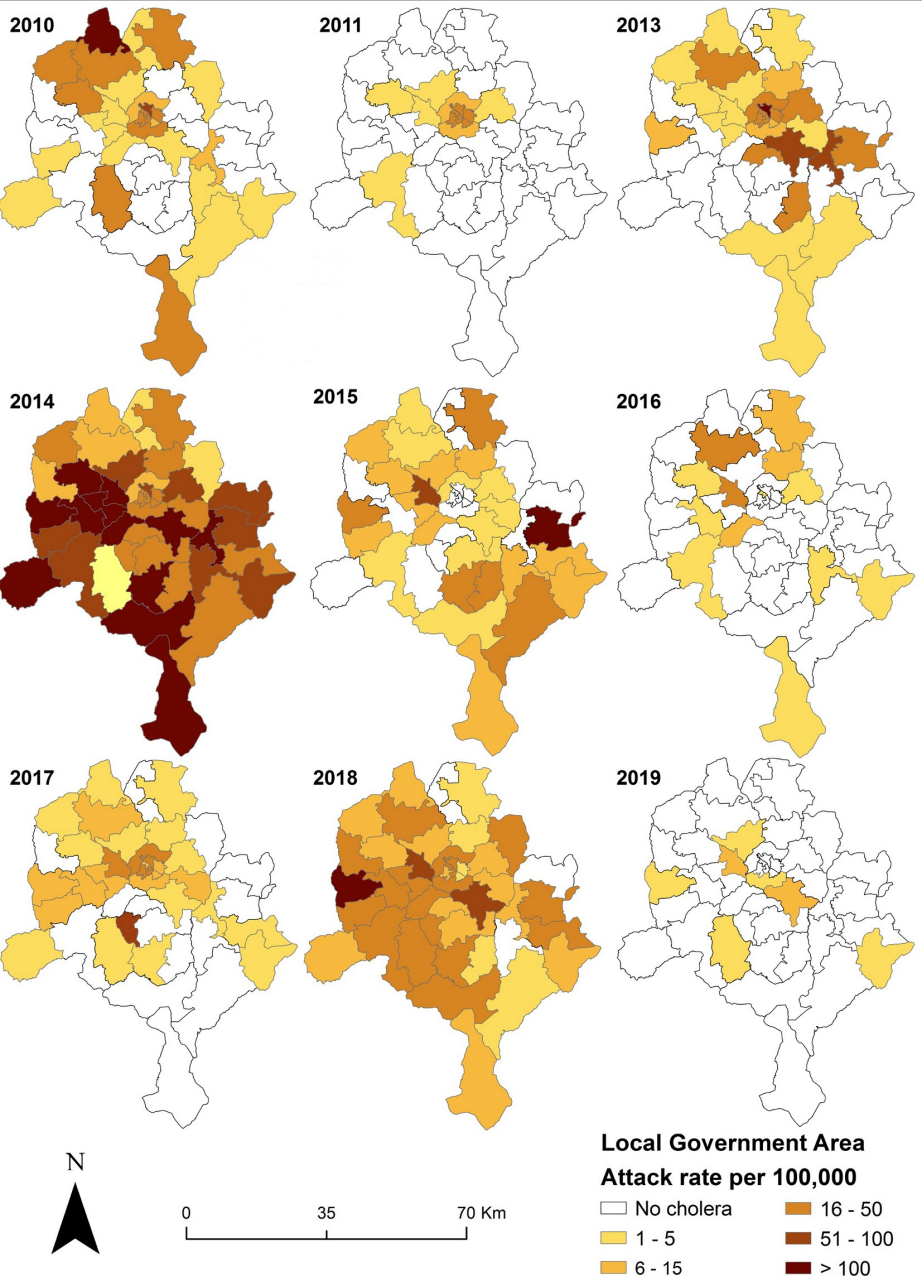
Geographic distribution of cholera attack rate at the Local Government Area (LGA) administrative level in Kano State from 2010–2019. No outbreaks were reported in 2012 while, cholera cases where reported from all LGAs in 2014.

To visualize spatial and temporal distribution and trends in the LGA-level incidence rates of reported cholera cases per 100 000 population (number of reported cholera cases during a year / annual population estimate), annual propagated epidemiological curve (Fig 2) and choropleth map (Fig 3) were produced for 2010 to 2019. Maps were produced using ArcGIS Version 10.5.1 (ESRI, Redlands, CA, USA).

### Cholera clusters/hotspots identification and classification analysis

To describe the space-time distribution of reported cholera cases across Kano State, we analyzed and described cholera clusters (hotspots) of yearly incidence at the LGA-level using two methods namely SaTScan and the GTFCC techniques. Firstly, we chose SaTScan because it is commonly used to identify clusters of disease cases [38] including cholera hotspots detection [16–18,39]; SaTScan detects hotspots by determining whether reported cholera cases in an LGA surpass what would be expected based on the LGA population size. Secondly, SaTScan incorporates spatial and spatio-temporal scan statistics with linkage to GIS for results visualization. We also used the GTFCC tool (S1 Data) [40], which is relatively new, and to assess how it compares with SaTScan in hotspots identification and classification. The GTFCC tool, which is based on Microsoft Excel, can readily be used with ease in resource poor settings at the periphery level (LGA and health facility level) by health personnel without statistical/GIS skill competencies needed to use SaTScan and GIS.

### Cholera cluster/hotspot identification by SaTScan (statistical) method

Clusters (hotspots LGAs) of cholera reported cases were identified using SaTScan v.9.6 through a retrospective analysis of the data for the ten-year period. To assess the dynamics of cholera clusters in the 10-year period, such as variations in size and duration, how clusters moved through space and time, we implemented a Poisson discrete space-time scan statistic following Kulldorf *et al*. [41]. In this vein, we scanned for high rates with 25% spatial and temporal windows, respectively, and default settings for all other values. The data used in this analysis were annual reported cholera cases for the ten-year period and the population of the LGAs for each year. The space-time cluster dynamics were presented in a table and visualized using ArcGIS Version 10.5.1 (ESRI, Redlands, CA, USA). The most likely cluster (hotspots LGAs) is the area that is least likely to have occurred by chance. In keeping with our objective of comparing hotspots detection using SaTScan and the newly developed GTFCC tool, the relative risks greater than 1 of the spatio-temporal scan statistic with p-value of 5% or less were shown in a table and mapped using ArcGIS Version 10.5.1 (ESRI, Redlands, CA, USA).

### Cholera hotspot identification as recommended by GTFCC

In September 2019, the GTFCC published new guidelines to be used by countries for the identification and classification of cholera hotspots or priority areas for intervention as part of their National Cholera Plan for cholera control or elimination [42]. Applying these guidelines, cholera hotspots were identified and ranked based on two epidemiological indicators namely, 1) mean annual incidence per 100 000 population of reported cases, and 2) the persistence of cholera for the study period. We excluded LGAs reporting less than or equal to two cases per 100 000 per year as these were considered sporadic cases without evidence of transmission. To determine the indicator mean annual incidence, we first determined the annual incidence by dividing the number of reported cholera cases of each LGA by its population each year. Next, the mean of the annual incidences for the ten-year study period was calculated for each LGA. To determine the indicator persistence, we divided the number of years with reported cholera cases by total number of years in the study period (i.e., 10 years). These two indicators were prepared using the GTFCC tool for identification of cholera hotspots in Microsoft Excel 2016 (S1 Data) [40].

Types of hotspot (T) were assigned to each LGA based on the two indicators delineating priority levels into high (T1), medium (T2), medium-low (T3), and low (T4). A 50^th^ percentile value was defined as the cut-off points for high mean incidence rate and persistence; however, the cut-off levels could be adjusted as desired. T1 hotspots corresponded to high priority areas and were LGAs with high mean annual incidence and high persistence of cholera during the surveillance period. T2 hotspots corresponded to medium priority areas, which were LGAs with high mean annual incidence rate and low persistence of cholera. T3 hotspots corresponded to medium-low priority LGAs characterized by low mean annual incidence rate, but a high persistence of cholera. T4 hotspots corresponded to low priority LGAs, which had low mean annual incidence rate and low persistence of cholera. The cut-off values for incidence and persistence delineating priorities/types of hotspot were chosen based upon the objectives of the NSPACC for Nigeria for 2018–2023 and resources available for effective implementation of intervention measures. Cholera hotspots classification chart and map were produced using the R statistical computing environment (version 3.6.2) [43] and Arc GIS, respectively. S2 Data contain the dataset underlying all analysis presented in this study.

## Results

### Descriptive spatio-temporal analysis

In our ten-year period under study, 16,461 cases of cholera were reported by the surveillance system amongst which 547 died (CFR 3.32%). In this time period, the mean annual incidence rate was 13.4 cases per 100 000 population with a mean annual mortality rate of 0.45 deaths per 100 000 individuals. Between 2010 and 2019, the most severe cholera exacerbations occurred in 2014 and 2018 when the annual incidence rates were 58.01 and 21.52 cases per 100 000 inhabitants, respectively (Fig 2). In 2018, cholera reported cases and deaths increased by 215% and 407%, respectively, compared to 2017. The highest case fatality ratios occurred in 2010 (CFR 4.98), 2011 (CFR 5.53) and 2018 (CFR 5.10). There was a decline in cases with no deaths reported in 2019 (Fig 2).

The distribution of LGAs with high reported cholera cases revealed considerable spatial heterogeneity between 2010 and 2019 (Fig 3). Excepting 2011 and 2019 where cholera cases concentrated in the central urban LGAs of the state, a majority of reported cases for the other years occurred in the rural LGAs of the north, south, east, and west portions. The widest geographic distribution of cases occurred during 2014 when all LGAs were affected, whereas, in 2018 cholera occurred in all LGAs except three in the north east (Fig 3).

### Cholera clusters/hotspots identification and classification

#### SaTScan space-time cluster detection

**Fig 4:**
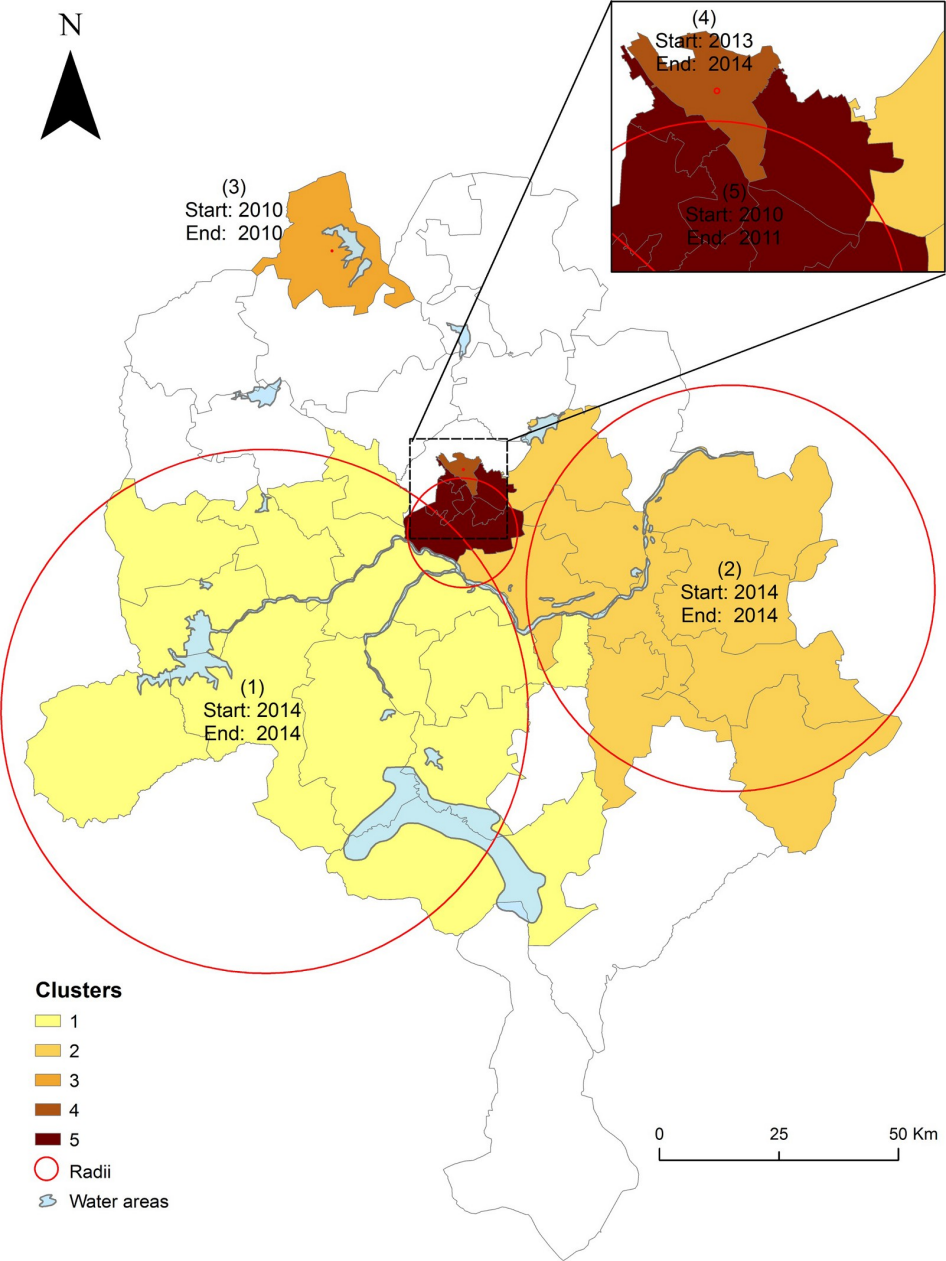
Spatio-temporal clusters of reported cholera cases in Kano State, 2010–2019. Map visualizes the space-time dynamics of reported clusters, which varied considerably in magnitude (size) and duration (time). Numbers 1–5 indicate clusters. Clusters 4 and 5 involve urban areas while the rest are located in the rural areas. The big circle shows a (1) large cluster with radius of 55.09 Km spanning 14 LGAs while the small circle indicates a (2) cluster of radius 42.72 Km involving nine LGAs. The smallest circle depicts a (5) cluster with radius of 11.49 Km involving six LGAs; the rest of the clusters (two LGAs) had zero radius, as indicated by the dots (red). Analysis used space-time windows of 25% of the population at-risk of cholera and 25% of the study time period, respectively, and default settings elsewhere. Here cholera cluster/hotspot means a geographically limited area where environmental, cultural and/or socioeconomic conditions facilitate the transmission of the disease and where cholera persists or reappears regularly. The insert shows a better view of the urban area clusters.

Between 2010 and 2019, results of SaTScan cluster detection yielded five statistically significant space-time clusters of reported cholera cases affecting 31 LGAs (Fig 4), as depicted in Table 1. The five clusters varied considerably in space and time within Kano State. One cluster was reported in 2010 with center in (3) Kunchi in the north and another between 2010 and 2011 with center in (5) Kumbotso in the Center. Between 2013–2014, a cluster was reported with center in (4) Fagge in the Center while during 2014, two large clusters were reported having centers in (1) Kiru in the west and (2) Gaya in the east, respectively. In the five reported clusters, excepting cluster 3, the least observed cases (clusters 4 and 5) involved LGAs in the urban portion of the state whereas the most observed cases (cluster 1 and 2) stem from LGAs in rural settings (Fig 4). Clusters 3 and 4 have 0 Km radius implying the two were contained within Kunchi and Fagge LGAs, respectively. In our study time period, clusters 4 and 5, located in urban settings, had longer durations (2 years) compared to those in the rural areas; duration of rural area clusters lasted only a year (Fig 4 and Table 1).

**Table 1:** Cholera clusters.

Cluster number	Duration (years)	Number of LGAs	P-value	Observed cases	Expected cases	Relative Risk (RR)	Population
1	2014–2014	14	1.0x10^-17^	2874	398	8.55	3100575
2	2014–2014	9	1.0x10^-17^	2132	302	7.97	2352684
3	2010–2010	1	1.0x10^-17^	303	17	18.37	149083
4	2013–2014	1	1.0x10^-17^	446	67	6.78	267000
5	2010–2011	6	1.0x10^-17^	1258	695	1.88	3035390
**Total**	**–**	**31**	**–**	**7013**	**1478**	**–**	**8,904,732**

**Fig 5:**
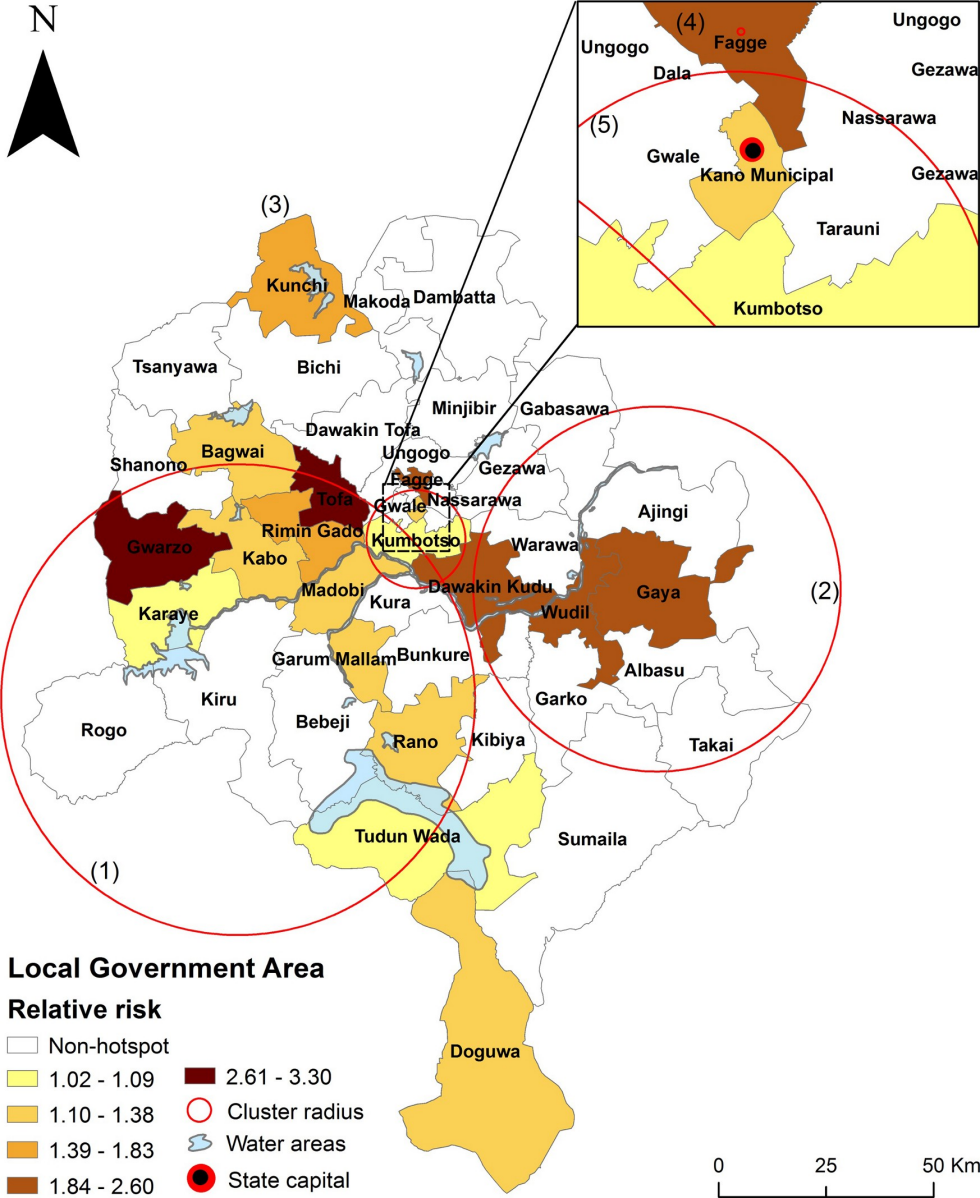
Statistically significant cholera hotspots Local Government Areas (LGAs) in Kano State, 2010–2019. Map depicts statistically significant relative risk (RR) per LGA for the cholera clusters (hotspot LGAs), i.e., LGAs within clusters with RR > 1. The LGA’s specific RR was calculated within each cluster using space-time scan statistics incorporating reported cholera cases and the population of the LGA for each year. Here cholera hotspot LGA means a geographically limited area where environmental, cultural and/or socioeconomic conditions facilitate the transmission of the disease and where cholera persists or reappears regularly. The insert shows a better view of the three urban hotspot LGAs.

Fig 5 and Table 2 show the relative risk (RR) greater than 1 for each LGA belonging to a significant space-time cluster of cholera between 2010 and 2019; and thus, the so-called hotspot LGAs. Of the 31 LGAs (out of 44) assigned to a cluster (Fig 4), 18 yielded relative risk greater than 1 (greater observed than expected cases) (Fig 5). Following is a breakdown of the 18 hotspots LGAs of RR > 1 (Fig 5 and Table 2). The (1) largest cluster in the western portion of the state spanned 11 hotspot LGAs, the (2) larger cluster in the east contains three hotspot LGAs while the large cluster at the Center involves two hotspots LGAs, and the ((4), (5)) lone clusters in the north and Center has 1 LGA each. Furthermore, the elevated cholera burden (hotspots LGAs) clustered significantly around water bodies, contextual factors of cholera transmission, including lakes and rivers (Fig 5). The relative risks of the 18 statistically significant hotspots LGAs ranged from 1.02 (Karaye) to 3.30 (Gwarzo) with a total population of 4,894,144 (Table 2). Six LGAs with a total population of 1.665 million had a relative risk greater than 2 compared to the state as a whole.

**Table 2:** Cholera hotspots classification based on statistical method (SaTScan).

LGA	Cluster number	p-value	Observed cases	Expected cases	Relative Risk (RR)*	Population
Gwarzo	1	1.0 x10^-17^	1020	323	3.30	280603
Tofa	1	1.0 x10^-17^	506	171	3.01	149057
Wudil	2	1.0 x10^-17^	820	325	2.60	282436
Dawakin Kudu	2	1.0 x10^-17^	910	395	2.38	343746
Fagge	4	1.0 x10^-17^	804	349	2.37	303237
Gaya	2	1.0 x10^-17^	792	353	2.31	306574
Rimin Gado	1	1.0 x10^-17^	334	184	1.83	159818
Kunchi	3	1.0 x10^-17^	348	195	1.80	169316
Rano	1	1.0 x10^-17^	349	255	1.38	221813
Kano Municipal	5	1.0 x10^-17^	829	641	1.31	557470
Kabo	1	1.0 x10^-17^	341	270	1.27	234607
Doguwa	1	1.0 x10^-17^	335	265	1.27	230570
Bagwai	1	1.0 x10^-17^	353	286	1.24	248361
Madobi	1	1.0 x10^-17^	295	240	1.24	208367
Garum Mallam	1	1.0 x10^-17^	243	204	1.19	177667
Kumbotso	5	1.0 x10^-17^	565	519	1.09	451404
Tudun Wada	1	1.0 x10^-17^	442	407	1.09	353434
Karaye	1	1.0 x10^-17^	253	248	1.02	215663
**Total**	**18**	**–**	**9539**	**5629**	**–**	**4,894,144**

*Note: Table is arranged in descending order of relative risk (RR), which represents the cholera incidence rate among the population in the scan window (exposed group), divided by the cholera incidence rate among the population out of the scan window (unexposed group). The LGA population reflects the year 2019 estimates.

### Cholera hotspot identification as recommended by GTFCC

**Fig 6:**
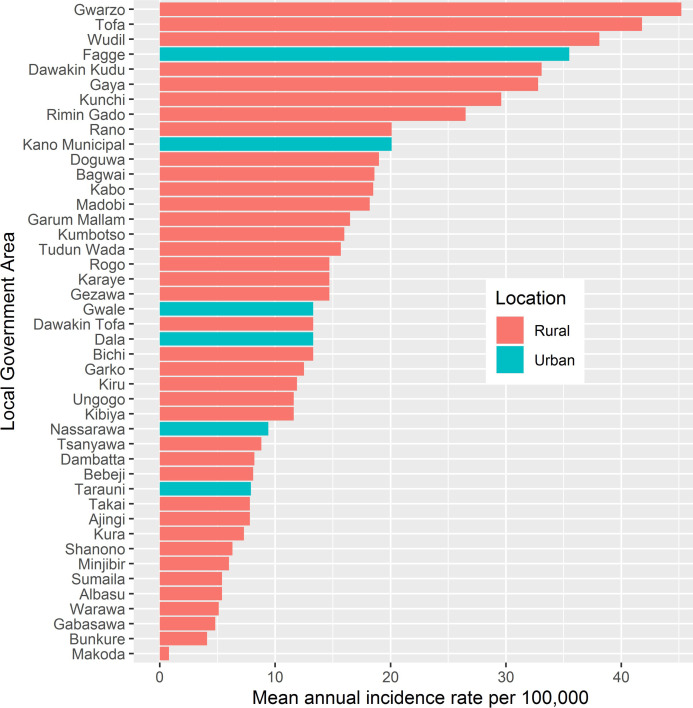
Distribution of mean annual incidence of cholera by urban and rural Local Government Areas (LGAs), 2010–2019. From top to bottom, the top three ranked LGAs with highest burden of cholera are rural LGAs (Gwarzo, Tofa and Wudil) while the first urban LGA (Fagge) with elevated burden of cholera comes at the fourth position. In the ten years of data analyzed, cholera occurs predominantly in rural 75% (6/44) compared to urban settings.

**Fig 7:**
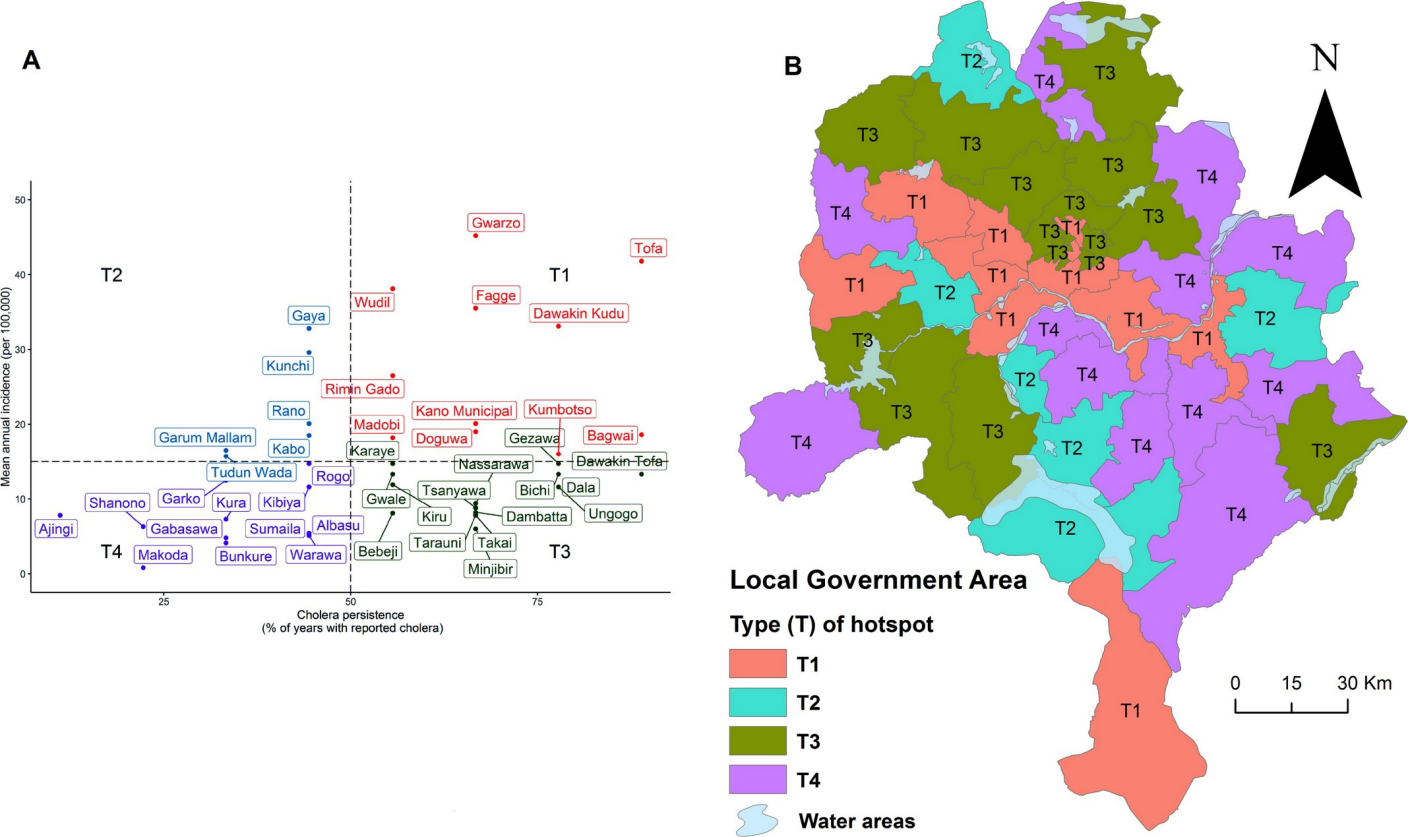
Cholera hotspot claissification chart (A) and map (B) for Kano State following GTFCC method using data from 2010–2019. **(A)** The persistence cut-off point is 50% and the mean annual incidence rate cut-off point is 15/100,000 population. T represents types of hotspot. T1 (high priority) reflects high mean annual incidence and high persistence of cholera; T2 (medium priority) shows hign mean annual incidence and low persistence of cholera; T3 (medium-low priority) indicates low mean annual Incidence and high persistence of cholera; and T4 (low priority) depicts low mean annual incidence and low persistence of cholera, respectively. The dots denote Local government Areas (LGAs) in the four types of hotspot quadrants. Compared with the statistical map in Fig 5, this GTFCC method misses seven LGAs in the T1 quadrant namely Kunchi, Kabo, Karaye, Gaya, Rano, Garum Mallam, and Tudun Wada, which, excepting Karaye, are in T2. However, this could be the result of the cut-off point. **(B)** Spatial map of the T (types of hotspots) in relation to permenent inland water areas/bodies.

In our study period, the LGAs with the highest mean annual incidence rates for cholera were the rural LGAs (Gwarzo, Tofa and Wudil) and the urban LGA (Fagge) (Fig 6). During the ten years of data analyzed, cholera occurred predominantly in rural, 75% (6/44), compared to urban settings. The mean annual incidence rate for each LGA that reported cholera ranged from 0.08 in Makoda to 45.20 per 100,000 persons in Gwarzo (Figs 6 and 3). Based on the 50^th^ percentiles, the cut-off for the mean annual incidence rate and persistence were 15/100,000 persons and 50%, respectively. Using these criteria, of the 44 LGAs in Kano State, 11 LGAs were identified as a T1 hotspot, 6 as a T2 hotspot, 15 as a T3 hotspot, and 12 as a T4 hotspot (Table 3). The cholera hotspot classifications chart and map (Fig 7A and 7B) are shown in Fig 7, and the list of the LGAs under the different categories of hotspots are provided in the Table 3. Further, populations of these hotspots LGAs are 3,215,067 for T1; 1,463,412 for T2; 6,333,534 for T3; and 3,299,251 for T4. Interestingly, five LGAs were identified as the top ranked five LGA hotspots using both methods (Tables 2 and 3). Still, excepting Karaye and considering T1 and T2 LGAs together, both methods converge in identifying 17 LGAs as high-risk cholera hotspots LGAs with a population of 4.678 million (Tables 2 and 3).

**Table 3:** LGAs with the type of hotspots based on mean annual incidence rate and proportion of years with reported cholera cases.

Type of hotspot (T)	LGA	Population	Mean Annual Incidence/100,000^±^	Proportion of years with reported cholera (%)
T1	Gwarzo	280603	45.2	66.7
T1	Tofa	149057	41.8	88.9
T1	Wudil	282436	38.1	55.6
T1	Fagge	303237	35.5	66.7
T1	Dawakin Kudu	343746	33.1	77.8
T1	Rimin Gado	159817	26.5	55.6
T1	Kano Municipal	557470	20.1	66.7
T1	Doguwa	230570	19.0	66.7
T1	Bagwai	248362	18.6	88.9
T1	Madobi	208367	18.2	55.6
T1	Kumbotso	451404	16.0	77.8
	**T1 Total**	**3,215,067**	**–**	**–**
T2	Gaya	306574	32.8	44.4
T2	Kunchi	169316	29.6	44.4
T2	Rano	221813	20.1	44.4
T2	Kabo	234607	18.5	44.4
T2	Garum Mallam	177668	16.5	33.3
T2	Tudun Wada	353435	15.7	33.3
	**T2 Total**	**1,463,412**	**–**	**–**
T3	Gezawa	430190	14.7	77.8
T3	Karaye	215663	14.7	55.6
T3	Bichi	422610	13.3	77.8
T3	Dala	638686	13.3	77.8
T3	Dawakin Tofa	378040	13.3	88.9
T3	Gwale	552184	13.3	55.6
T3	Kiru	403823	11.9	55.6
T3	Ungogo	563772	11.6	77.8
T3	Nassarawa	909993	9.4	66.7
T3	Tsanyawa	240482	8.8	66.7
T3	Dambatta	317177	8.2	66.7
T3	Bebeji	288033	8.1	55.6
T3	Tarauni	337613	7.9	66.7
T3	Takai	309208	7.8	66.7
T3	Minjibir	326062	6.0	66.7
	**T3 Total**	**6,333,534**	**–**	**–**
T4	Rogo	347335	14.7	44.4
T4	Garko	247833	12.5	33.3
T4	Kibiya	208539	11.6	44.4
T4	Ajingi	265580	7.8	11.1
T4	Kura	220534	7.3	33.3
T4	Shanono	214443	6.3	22.2
T4	Albasu	290007	5.4	44.4
T4	Sumaila	386864	5.4	44.4
T4	Warawa	196416	5.1	44.4
T4	Gabasawa	321885	4.8	33.3
T4	Bunkure	260630	4.1	33.3
T4	Makoda	339186	0.8	22.2
	**T4 Total**	**3,299,251**	**–**	**–**

± Note: Table is arranged in descending order of mean annual incidence within the hotspots types (T). The Local Government Area (LGA) population reflects the year 2019 estimates.

## Discussion

This study analyzed cholera disease frequencies, distribution patterns, and hotspots that were associated with disease transmission in Kano State, Nigeria, from 2010 to 2019. In this time period, annual epidemics waxed and waned in Kano State from year to year with considerable heterogeneous geographical distribution. While Kano State is considered a state with high rates of cholera, the risk of cholera transmission within the different LGAs is heterogeneous. This study identified several hotspot LGAs that were at higher risk compared to other LGAs and the two methods used were generally in agreement as to the identification of these LGAs. Excepting the three urban hotspots LGAs where population density seem to drive cholera hotspots [11], we found that the rural hotspots LGAs tend to cluster around major water bodies. Yet, more data (e.g., WASH and socio-economic data) is needed to better understand differences between the urban and rural settings that contribute to differential cholera outcomes. In keeping with our main objective of providing actionable information to the NSPACC to guide cholera interventions between 2020 and 2023, the T1 LGAs in Table 3 would appear to be high priority for vaccination by the NCDC, National Primary Health Care Development Agency (NPHCDA) and Kano State Ministry of Health while longer term WASH infrastructure is being put in place in line with the global roadmap of ending cholera by 2030.

Excepting the year 2012, cholera yearly exacerbation and heterogeneous Spatial distribution in Kano State are consistent with findings in other settings [17,44]. In our study period the CFR is high (>3%), far exceeding the less than 1% target as recommended by the WHO [45], a finding that has been documented in previous studies [24,25,33]. A number of factors underlie the high fatality ratios we found in this study including poor case management at health facilities, care seeking behavior, and burial traditions. The latter factor underscores the need for health system strengthening and personnel training while the former two factors call for community sensitization for members to seek immediate care at health facility at the first notice of symptoms of cholera. As spatio-temporal epidemiology of cholera in Cameroon documented [17], cholera outbreaks in Kano State seem to follow a 3–4 year life cycle with peaks in 2010, 2014, and 2018 (Fig 2). Although research on the contextual drivers of these outbreaks and peaks is scarce, public health authorities in Kano State blame poor socio-economic conditions, lack of adequate WASH systems as well as environmental (lakes, rivers) and climatic factors (precipitation) for the outbreaks and peaks. Still, why there was no cholera in 2012 and no deaths reported in 2019 are not well understood either. As such, studies are needed to address each of these observations vis-a-vis aforementioned contextual factors. There are plans to provide vaccine in Kano State during the coming years and this analysis should be helpful to preparing for the specific plans for vaccination. The population of LGAs with a significantly higher incidence was 4.894 million, or alternatively, the population of the LGAs with a relative risk of > 2 was 1.665 million. With respect to vaccines the population of these hotspots LGAs of about 4,894,144 (Table 2), make up 34.2% of the total population of Kano State. Five of the six LGAs with a relative risk > 2 (Gwarzo, Tofa, Wudil, Dawakin Kudu, and Fagge) were identified as T1 hotspots using the GTFCC method. The other LGA (Gaya) was identified as a T2 hotspot with a high mean annual incidence but lower persistence. Since the cut-off values for determining the T values are adjustable, local information might suggest moving this LGA higher into T1 priority.

As *V*. *cholerae*, the causative agent of cholera, thrives in aquatic environments our finding that the hotspot LGAs cluster around water bodies (Fig 5) is consistent with findings elsewhere of lakes and rivers as contextual factors linked with cholera transmission [17,20]. Earlier in 2017, a desk review which incorporated data from 2012–2017 was conducted for the NSPACC to estimate hotspot LGAs in Kano Sate [27]. Based on a threshold of mean annual incidence of ≥ 1 case per 100 000 population and stakeholder weighting, the desk review method identified 27 LGAs as hotspots with three high and seven medium priority ranked LGAs (S1 Table). By contrast, both the SaTScan and GTFCC methods we used identified 18 high priority hotspots LGAs (Tables 2 and 3). In addition, the existing map in the NSPACC identified Garko, Kibiya, Rogo, and Ajingi as hotspots LGAs (S1 Table), but neither SaTScan nor GTFCC methods classified these LGAs as hotspots. These differences could result from the fact that we used data for a longer time period (10 years) with rigorous statistical methods and ignored mean annual incidence ≤ 2 cases per 100 000 people while the desk review data spanned six years using mean annual incidence of 1 case per 100 000 inhabitants. As we found in this study, SaTScan has been widely used and found very helpful in identification and classification of cholera clusters (hotspots) to inform intervention measures in SSA including Cameroon [17], Uganda [18], Zambia [46], and Tanzania [47]. However, the GTFCC method is relatively new and we found no evidence of its use in published reports. The similarity of the results using the two methods is reassuring and the data from each may be useful to local policy makers who will need to include local factors with hotspots analysis when making decisions for finally determining priorities for interventions.

This study has important limitations as well as strengths. The surveillance data used in these analyses can underestimate or overestimate the true rates of cholera, as cases can be misclassified because most cases are based on clinical criteria and are not lab confirmed. Further, we used annual data in this study whereas space-time scan statistic and “Persistence” in conjunction with SaTScan and GTFCC methods, respectively, are better if they can be based on weekly or monthly data. Nonetheless, use of the national surveillance data based on IDSR strategy over a long period of time offered the opportunity to describe hotspots LGAs needed to inform intervention measures including vaccine; and thus, constitute major strengths of the study. Another strength is the use of two methods to cluster identification, which show SaTScan and GTFCC in agreement vis-à-vis high risk (hotspots) LGAs. Although SaTScan is widely used in cluster detection, its use demands a strong statistical background knowledge compared to the GTFCC method, which demands only the ability to enter data into Microsoft Excel. As such, the later method can readily be applied in resource poor settings without statisticians for hotspots identification.

Although we observed qualitatively that cholera risk clustered around waterbodies, we did not quantify the magnitude of this association. The quantification of this association would have enriched this study, and quantification of this contextual factor beside others (rainfall, access to care, care-seeking behavior, and burial traditions) and WASH indicators would be very critical in future studies. In other countries, fishing villages have a higher risk [19] but this information was not available for this study. Most importantly, future work will need to refine these hotspots to a finer level of wards to identify micro-hotspots within the LGAs, but other methods will be needed to handle the problem of unstable rates due to small population sizes. In addition, associations between socioeconomic and WASH indicators correlate poorly when larger populations (e.g. entire LGAs) are used [18,48], and so exploring these associations at ward level in future efforts will fill a critical gap in literature and help identify directions needed for WASH interventions. Importantly, hotspot analysis like this need to be integrated with local knowledge when planning for vaccination campaigns and WASH interventions.

Conclusively, in 2017, GTFCC called for focusing cholera interventions in cholera hotspots [10]. In 2018, Nigeria launched the NSPACC to pre-emptively vaccinate populations in identified hotspots nationwide between 2018 and 2023 [27]. We believe that our study identifies high priority hotspot LGAs in Kano State to guide NSPACC’s cholera interventions including vaccine towards ending cholera in the state by 2030.

## Supporting information

S1 Data The GTFCC tool for identification of cholera hotspots. (XLSX)

S2 Data Data underlying all figures. (XLSX)

S1 Table Table comparing Desk Review method of cholera hotspots classification with SaTScan and GTFCC methods. *The current hotspot in the Nigeria National Strategic Plan of Action on Cholera Control (NSPACC) is based on the desk review method, which have more LGAs than SaTScan and GTFCC methods. We note that Garko, Kibiya, Rogo, and Ajingi are not hotspots LGAs according to SaTScan and GTFCC methods and that Gwarzo and Tofa classified by Desk review as medium priority and the top most priority (T1) following SaTScan and GTFCC. In addition, six out of the 15 LGAs classified as low priority by the Desk Review are high priority (T1) according to SaTScan and GTFCC.
(DOCX)
