# Supplementary material for: The cholera risk assessment in Kano State, Nigeria: A historical review, mapping of hotspots and evaluation of contextual factors
Source: PLoS Negl Trop Dis. 2021 Jan 19;15(1):e0009046. doi: 10.1371/journal.pntd.0009046 (PMC7846125; doi:10.1371/journal.pntd.0009046)
Supplement: S1 Table — *The current hotspot in the Nigeria National Strategic Plan of Action on Cholera Control (NSPACC) is based on the desk review method, which have more LGAs than SaTScan and GTFCC methods. We note that Garko, Kibiya, Rogo, and Ajingi are not hotspots LGAs according to SaTScan and GTFCC methods and that Gwarzo and Tofa classified by Desk review as medium priority and the top most priority (T1) following SaTScan and GTFCC. In addition, six out of the 15 LGAs classified as low priority by the Desk Review are high priority (T1) according to SaTScan and GTFCC. (DOCX) [file pntd.0009046.s003.docx]

**Supplementary material**

**S2 Supplement: Table comparing Desk Review method of cholera hotspots classification with SatScan and GTFCC methods.**

| **State** | **LGAs** | **Desk review method*** | **SatScan method** | **GFTCC method** |
| --- | --- | --- | --- | --- |
| KANO | Dawakin Kudu | High | 1 | T1 |
| KANO | Tudun Wada | High | 1 | T2 |
| KANO | Doguwa | High | 1 | T1 |
| KANO | Gwarzo | Medium | 1 | T1 |
| KANO | Tofa | Medium | 1 | T1 |
| KANO | Gwale | Medium |  | T3 |
| KANO | Kabo | Medium | 1 | T2 |
| KANO | Bagwai | Medium | 1 | T1 |
| KANO | Bichi | Medium |  | T3 |
| KANO | Garko | Medium |  |  |
| KANO | Kibiya | Medium |  |  |
| KANO | Takai | Medium |  | T3 |
| KANO | Wudil | Low | 1 | T1 |
| KANO | Gaya | Low | 1 | T2 |
| KANO | Gezawa | Low |  | T3 |
| KANO | Rogo | Low |  |  |
| KANO | Dawakin Tofa | Low |  | T3 |
| KANO | Ungongo | Low |  | T3 |
| KANO | Rano | Low | 1 | T2 |
| KANO | Madobi | Low | 1 | T1 |
| KANO | Karaye | Low | 1 | T2 |
| KANO | Ajingi | Low |  |  |
| KANO | Garum Mallam | Low | 1 | T2 |
| KANO | Fagge | Low | 1 | T1 |
| KANO | Kano Municipal | Low | 1 | T1 |
| KANO | Rimin Gado | Low | 1 | T1 |
| KANO | Kumbotso | Low | 1 | T1 |

*The current hotspot in the Nigeria National Strategic Plan of Action on Cholera Control (NSPACC) is based on the desk review method, which have more LGAs than SatScan and GTFCC methods. We note that Garko, Kibiya, Rogo, and Ajingi are not hotspots LGAs according to SatScan and GTFCC methods and that Gwarzo and Tofa classified by Desk review as medium priority and the top most priority (T1) following SaTScan and GTFCC. In addition, six out of the 15 LGAs classified as low priority by the Desk Review are high priority (T1) according to SaTScan and GTFCC.
